# Migrating the SNP array-based homologous recombination deficiency measures to next generation sequencing data of breast cancer

**DOI:** 10.1038/s41523-018-0066-6

**Published:** 2018-07-02

**Authors:** Zsofia Sztupinszki, Miklos Diossy, Marcin Krzystanek, Lilla Reiniger, István Csabai, Francesco Favero, Nicolai J. Birkbak, Aron C. Eklund, Ali Syed, Zoltan Szallasi

**Affiliations:** 1 Department of Bio and Health Informatics, Technical University of Denmark, Kemitorvet 208, Lyngby, 2800 Denmark; 2 1st Department of Pathology and Experimental Research, Semmelweis University, Budapest, Hungary; 3 2nd Department of Pathology, MTA-SE NAP, Brain Metastasis Research Group, Hungarian Academy of Sciences, Semmelweis University, Budapest, Hungary; 4 Department of Physics of Complex Systems, Eötvös Loránd University, Budapest, Hungary; 5 Biotech Research & Innovation Centre, University of Copenhagen, Ole Maaløes Vej 5, Copenhagen, DK-2200 Denmark; 6 The Francis Crick Institute, London, UK; 7 University College London Cancer Institute, London, UK; 8 Danish National Life Science Supercomputing Center, Copenhagen, Denmark; 9 Computational Health Informatics Program, Boston Children’s Hospital, Boston, MA USA; 10 Harvard Medical School, Boston, MA USA

## Abstract

The first genomic scar-based homologous recombination deficiency (HRD) measures were produced using SNP arrays. As array-based technology has been largely replaced by next generation sequencing approaches, it has become important to develop algorithms that derive the same type of genomic scar scores from next generation sequencing (whole exome “WXS”, whole genome “WGS”) data. In order to perform this analysis, we introduce here the scarHRD R package and show that using this method the SNP array-based and next generation sequencing-based derivation of HRD scores show good correlation (Pearson correlation between 0.73 and 0.87 depending on the actual HRD measure) and that the NGS-based HRD scores distinguish similarly well between BRCA mutant and BRCA wild-type cases in a cohort of triple-negative breast cancer patients of the TCGA data set.

## Introduction

Reliable quantification of homologous recombination deficiency of human tumor biopsies, especially in the case of ovarian and breast cancer, is expected to identify patients that are particularly sensitive to platinum or PARP inhibitor-based therapy.^1^ Before the widespread introduction of next generation sequencing (NGS) to characterize tumor biopsies, SNP arrays were used to identify large-scale genomic aberrations associated with homologous recombination deficiency, often induced by the loss of BRCA1 or BRCA2 function. Three such measures were identified: telomeric allelic imbalance (HRD-TAI score),^2^ loss of heterozygosity profiles (HRD-LOH score),^3^ and large-scale state transitions (HRD-LST score).^4^ These three measures have also been combined into a single summary measure of HR deficiency.^5^ The HRD-LOH score has also become an integral part of a recently published, whole-genome sequencing-based measure of homologous recombination deficiency, HRDetect.^6^ These measures, along with functional assays,^7^ showed promise to identify HR-deficient cases and thus predict response to platinum or PARP inhibitor therapy.^2,8,9^ Since NGS has become the main genomic characterization method of cancer biopsies, it has become essential to migrate the SNP array-based methodology to NGS-based platforms.

TCGA breast cancer biopsies have been both SNP array profiled and subjected to NGS allowing a direct comparison.^8^

## Results and discussion

**Fig. 1:**
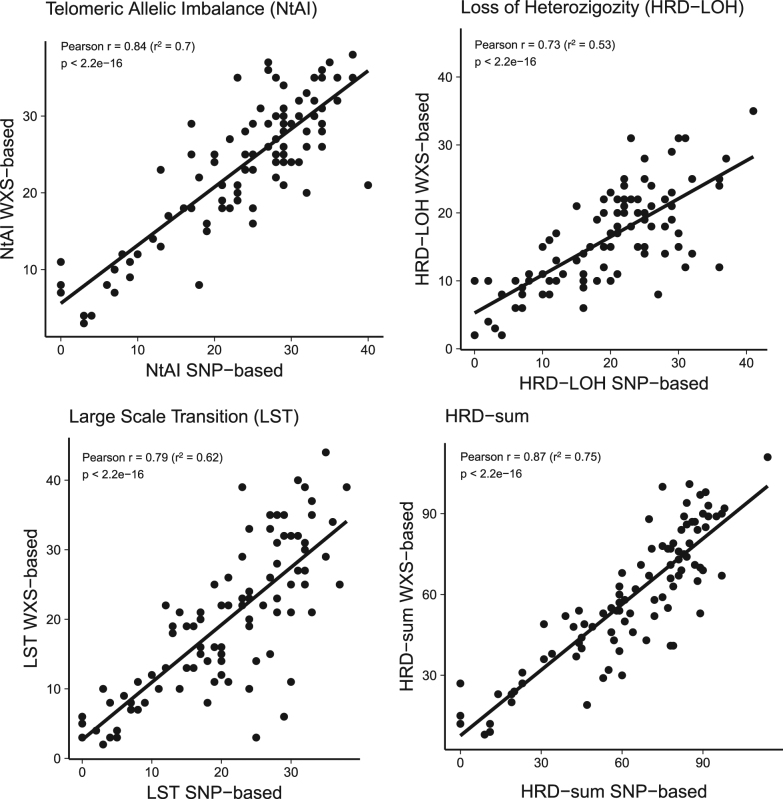
Correlation between Affymetrix SNP 6.0 array-based and whole exome sequencing-based measurements of homologous recombination deficiency (telomeric allelic imbalance, loss of heterozygosity, large-scale transitions, and the sum of these estimates)

**Fig. 2:**
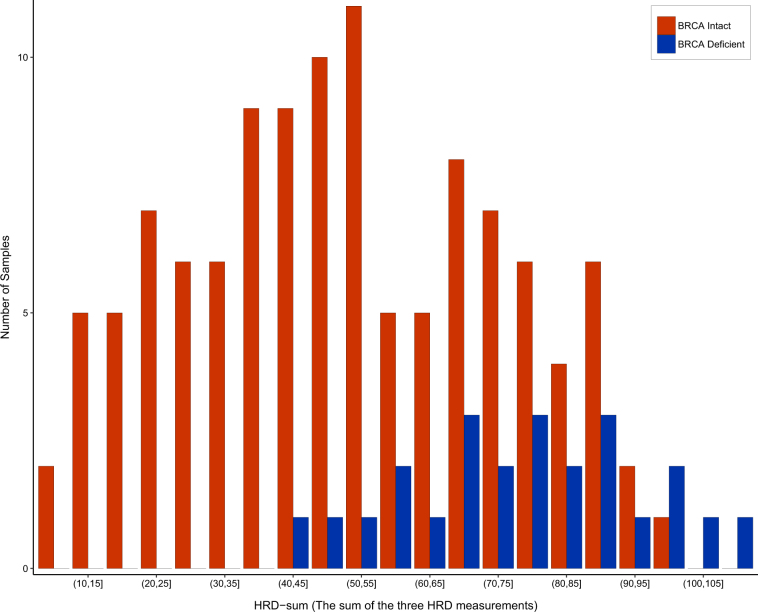
Distribution of HRD-sum values in BRCA1/2 deficient and in BRCA1/2 intact triple-negative breast cancer samples from TCGA. HRD-sum values were determined with the scarHRD R package

We found good correlation between the SNP array-based and NGS-based HRD scores (Fig. 1). When comparing the results of the scarHRD R package to SNP array-based measurements, we found the following Pearson correlation coefficients: number of telomeric allelic imbalances (NtAI): *r* = 0.84 (*R*^2^ = 0.70, adjusted *R*^2^ = 0.70, *p* < 2.2e–16), large-scale transition (LST) *r* = 0.79 (*R*^2^ = 0.62, adjusted *R*^2^ = 0.62, *p* < 2.2e–16) loss of heterozygosity (HRD−LOH) *r* = 0.73 (*R*^2^ = 0.53, adjusted *R*^2^ = 0.52, *p* < 2.2e–16). These three measures are often combined for diagnostic purposes^5^ and in HRDetect.^6^ Therefore, we also compared the sum of the three scores across the two platforms (HRD sum): *r* = 0.87 (*R*^2^ = 0.75, adjusted *R*^2^ = 0.75, *p* < 2.2e–16) (Fig. 1). The artificial reduction of coverage to 30× did not affect this correlation (Supplementary Material, Figure S7-S8). The BRCA1/2-mutated samples showed significantly higher NGS-based HRD-sum values (Fig. 2, Supplementary Figure S6). The predictive value of HRD-sum, measured as AUC value of the corresponding ROC curve, was 80.8% (Supplementary Figure S2).

There was no significant difference in SNP versus WXS-based estimation of tAI, LST, and HRD-sum, but the number of LOH events were significantly lower in the WXS-based estimation (*p* = 0.012, Kolmogorov–Smirnov test). This could be attributed to differences in segmentation algorithm (the more segmented the WXS data is the lower number of LOHs that are called) or to low sample quality, coverage. However, when comparing the ROC curves for BRCA1/2 status of the SNP-based and WXS-based HRD-score, there was no significant difference between the SNP array-based and NGS-based methods. (Supplementary Figure S3).

According to our expectations and previous results the BRCA1/2-deficient cases showed higher values for each of the four scores (Supplementary Figure S4-S5).

The sum of the three HRD scores showed good correlation across the two platforms. Thus in more advanced NGS-based HR deficiency measures such as HRDetect, the SNP array-based step could be replaced by an NGS-based estimate of the HR deficiency scores.

### Brief description of the methods

Based on receptor status determined by immunohistochemistry, 139 paired tumor and normal samples of the TCGA breast cancer cohort could be classified as triple-negative breast cancer. From these patients 95 had Affymetrix SNP 6.0 array-based HRD estimates (LOH, TAI, LST), previously published by our group.^10^ In this publication we present the scarHRD R package (https://github.com/sztup/scarHRD) which estimates the level of the three HR deficiency measures using NGS data.

A sample’s LOH score is the total number of LOH regions across the entire genome that are larger than 15 Mb but do not cover whole chromosomes. In the original publication this 15 Mb lower limit for LOH was determined by comparing SNP array profiles between BRCA mutant and BRCA wild-type cases.^3^ We performed a similar analysis using NGS data and found that the original 15 Mb cutoff performed best in this case as well (Supplementary Figure S1).

The LST is defined as a chromosomal break between adjacent regions of at least 10 Mb, with a distance between them not larger than 3 Mb.

The number of telomeric allelic imbalances is the number of AIs (the unequal contribution of parental allele sequences with or without changes in the overall copy number of the region) that extend to the telomeric end of a chromosome.

Allele-specific copy number estimation is a crucial part of estimating HR deficiency. As previously shown, allele-specific copy number estimation from NGS data performed using the Sequenza R package show high agreement with SNP array-based copy number profiles.^11^ The scarHRD package is, therefore, able to use Sequenza preprocessed files as well as other allele-specific segmentation files in the same format.

As it has been previously shown that in ovarian cancer the sum of the genomic scar scores is elevated in BRCA-deficient cancers,^5^ an additional aim of our study was to compare the unweighted numeric sum of LOH, tAI, and LST, called here HRD-sum, to the BRCA1/2 status of the patients. A sample was classified as BRCA-deficient if (1) there was a deep deletion of BRCA1/2, (2) a germline and a somatic mutation in BRCA1/2 with LOH, or (3) if LOH had co-occurred with promoter methylation in one of the *BRCA1/2* genes. The somatic mutation status (mutations with likely pathogenic function) and methylation data was acquired from the TCGA data portal. The germline mutation status was determined using HaplotypeCaller, and was annotated with Intervar,^12^ likely pathogenic mutations and frameshift insertion/deletion with unknown significance were used in our analysis. LOH was determined using Sequenza’s allele-specific segmentation results (Supplementary Table S1).

### Data availability

The data sets generated during the current study are available from the corresponding author on reasonable request.

### Code availability

The code/algorithm for performing the experiments is available for download at https://github.com/sztup/scarHRD.

## Electronic supplementary material

Supplementary Material
