# Supplementary material for: Migrating the SNP array-based homologous recombination deficiency measures to next generation sequencing data of breast cancer
Source: NPJ Breast Cancer. 2018 Jul 2;4:16. doi: 10.1038/s41523-018-0066-6 (PMC6028448; doi:10.1038/s41523-018-0066-6)
Supplement: Supplementary file 1 — Supplementary Material [file 41523_2018_66_MOESM1_ESM.pdf]

Supplementary Material

Supplementary Methods

Optimalization of the cut-off of the size of LOH regions

In order to find the most appropriate cutoff value for the minimum length of LOH to be considered in the HRD-LOH score we investigated that which cutoff-value based HRD-LOH score would be able to discriminate the BRCA-deficient, and intact samples the best. We considered using Mann-Whitney or Kolmogorov-Smirnov. (Both nonparametric tests to compare two unpaired groups of data.) Abkevich et al. [1] used Kolmogorov-Smirnov test, however Mann-Whitney is more sensitive to changes in the median of the groups, thus we carried out the analysis both with Kolmogorov-Smirnov test (**Supplementary Figure S1A** and Mann-Whitney test (**Supplementary Figure S1B**). In the Abkevich et al. paper the cut-off of 15 Mb was slightly arbitrarily selected, rather than selecting the cut-off with the lowest p-value (cut-off of 7 Mb). The authors argue that the rational for this selection rather than selecting the cut-off with the lowest p-value is that the 15 Mb cut-off is more sensitive to statistical noise present in the data.

We also performed Spearman rank correlation between the SNP-array-based and WXS-based HRD-LOH scores for the different cutoff minimum LOH lengths (**Supplementary Figure S1C**). Here the 14 Mb and 15 Mb cutoff-based WXS-HRD-LOH score had the highest correlation with the SNP-based HRD score. (0.700 and 0.695 respectively). This result reassured our choice of using the 15 Mb cutoff like in the SNP-array-based HRD-LOH score.

Predictive value of WXS-based HRD-sum

The predictive value of HRD-sum, measured as AUC value of the corresponding ROC curve, was 80.8% (**Supplementary Figure S2**).

The effect of WXS-based estimation of genomic scar scores on their discriminating value

In order to investigate if either genomic scar score individually deteriorates on WXS data, we compared the ROC-curves of BRCA-status predictive potential for each of the scar scores and for HRD-sum between the SNP-based and the WXS-based results. To plot the curves and test difference we used the pROC R package [2]. None of the WXS-based curves were significantly different from the SNP-array-based ones (DeLong's test, n = 95). (**Supplementary Figure S3A-D**)

Differences in the genomic scar score between BRCA-deficient and BRCA-intact samples

We investigated the differences between BRCA-deficient and BRCA-intact samples and all of the score were significantly elevated in the in BRCA-deficient group (p<0.001, Mann-Whitney test) **(Supplementary Figure S4A-D, Supplementary Figure S5)**

So we can represent the difference of HRD-sum distribution between BRCA Intact and Deficient samples we prepared a version of Figure 2, where the y-axis represents the fraction of the samples from the BRCA Intact or Deficient group instead of the number of patients. (**Supplementary Figure S6**)

Effect of coverage on the individual genomic scar scores

To evaluate the effect of coverage on the individual genomic scar scores, we determined the coverage of 15-15 randomly selected BRCA-deficient and BRCA-intact samples. The coverage was estimated using Mosdepth[3]. The Pearson correlation between the coverage and the genomic scars were low in each of the cases (**Supplementary Figure S7**). The HRD-LOH score showed the "highest" correlation ($r = 0.067$, $r2 = 0.0045$) with average of tumor-, normal depth. To further test the effect of coverage we simulated a reduced, 30x coverage for these samples selecting reads randomly using SAMtools for this subsampling[4]. (The original coverage of these 30 germline and tumor samples were 79x, 85x, respectively. Each of the genomic scar score showed high Pearson-correlation between the 30x (subsampled) and the original samples. (**Supplementary Figure S8**)

Supplementary Figure S1A

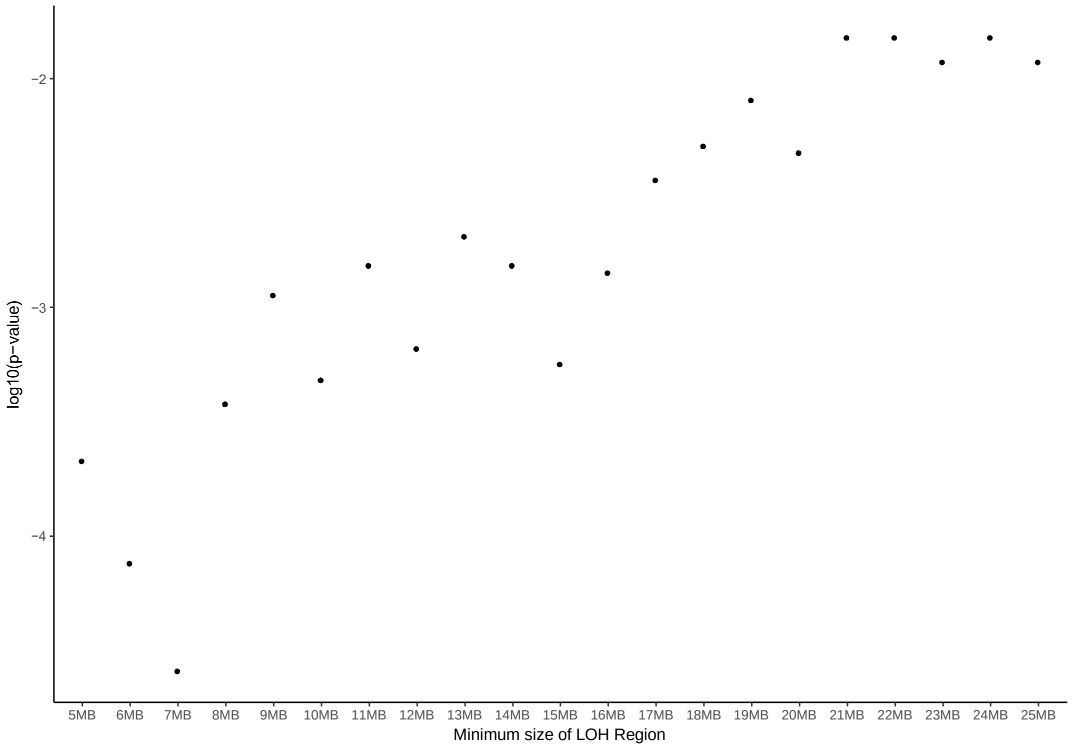

Supplementary Figure S1A: Optimalization of the cut-off of the size of LOH regions Correlation between HRD-LOH scores and BRCA1/2 deficiency was calculated for different LOH region length cut-offs. The corresponding log10(p-value) of the Kolmogorov–Smirnov test are on the y-axis. Since the original (SNP-based) 15 Mb cut off value was one of the most significant lower size limits we chose to use this in our further analysis.

Supplementary Figure S1B

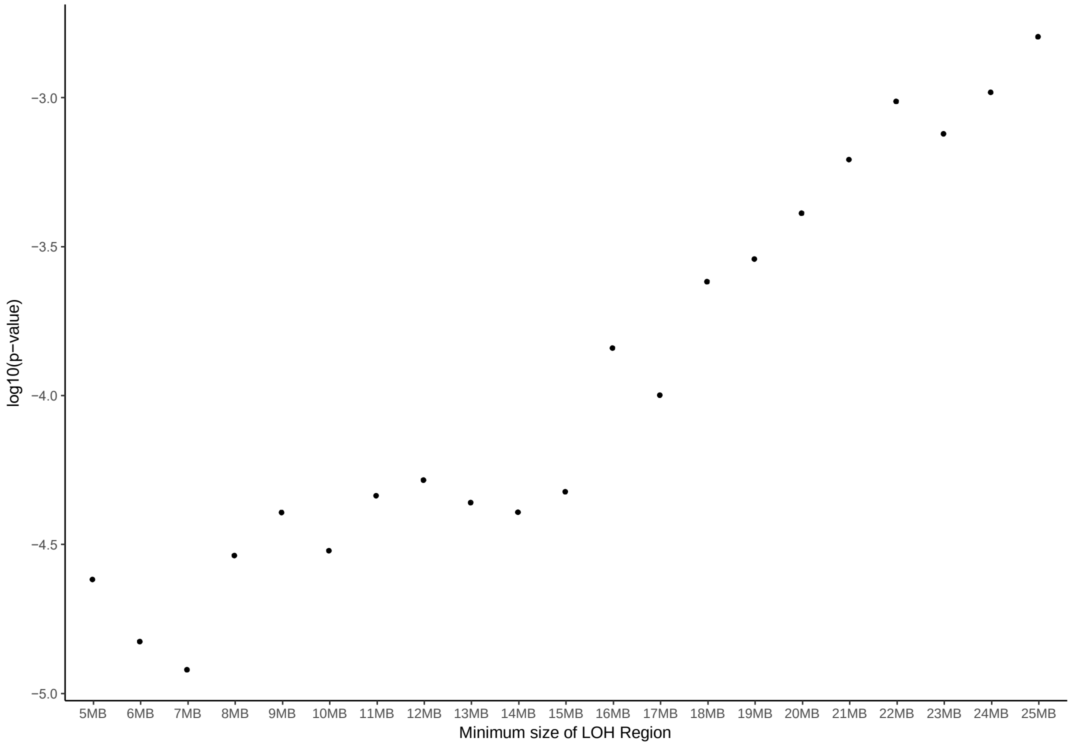

Supplementary Figure S1B: Optimalization of the cut-off of the size of LOH regions Correlation between HRD-LOH scores and BRCA1/2 deficiency was calculated for different LOH region length cut-offs. The orresponding log10(p-value) of the Mann-Whitney test are on the y-axis.

Supplementary Figure S1C

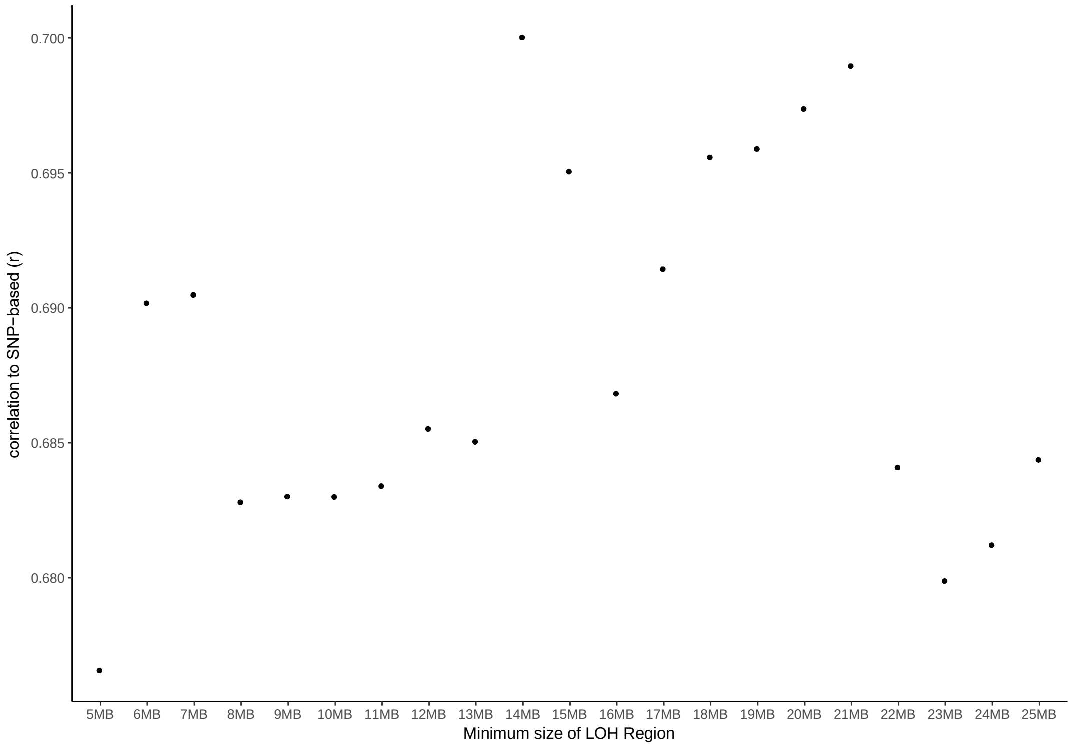

Supplementary Figure S1C: Spearman rank correlation between the SNP-array-based and WXS-based HRD-LOH scores for the different cutoff minimum LOH lengths (5 Mb to 25 Mb).

Supplementary Figure S2

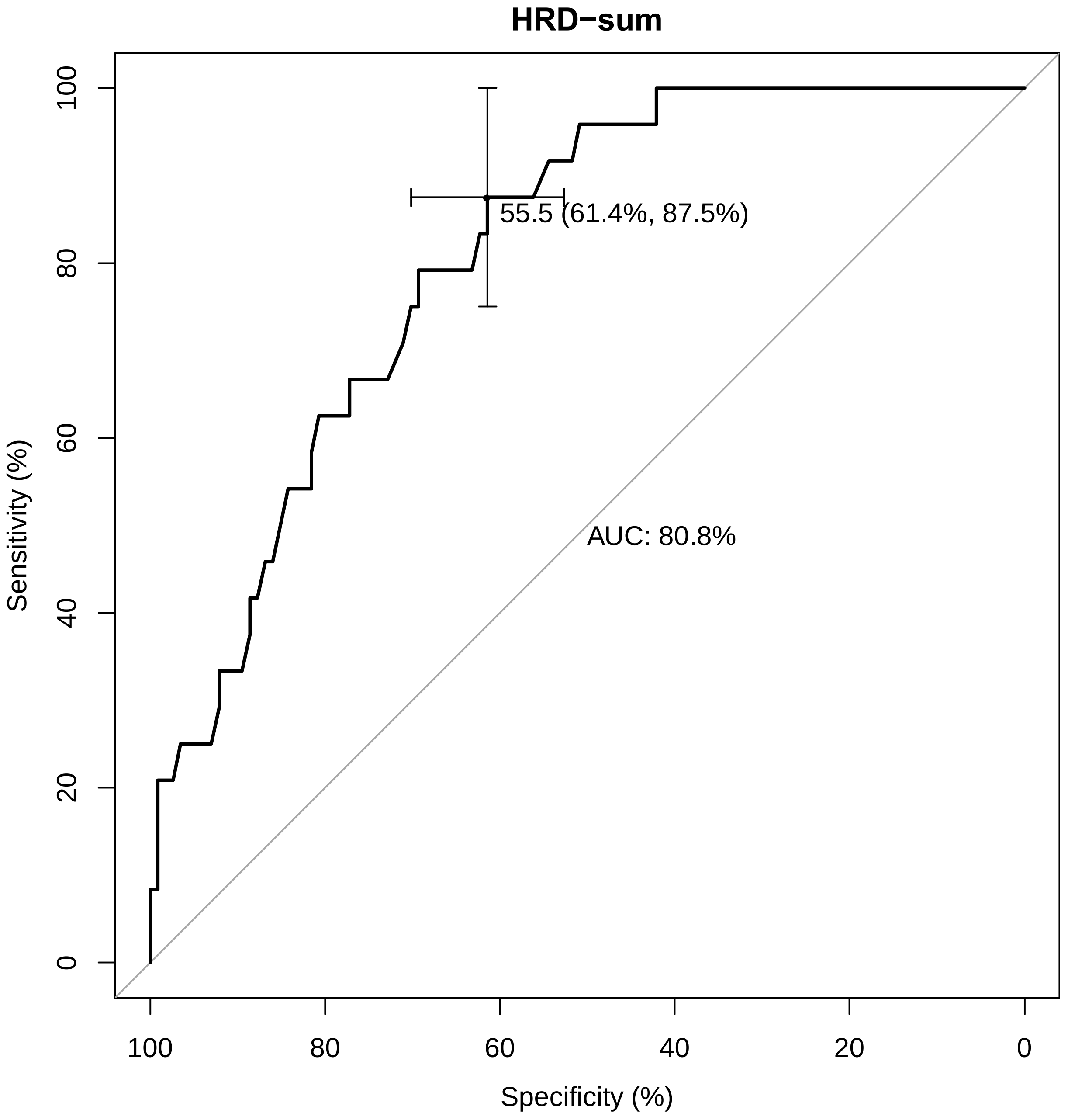

Supplementary Figure S2: ROC curve showing the ability of the WXS-based HRD-sum to predict the BRCA1/2 deficiency status in n=139 triple-negative breast cancer patients of the TCGA dataset. The error bar around the best cutoff value represents the 95% confidence interval of the sensitivity and specificity.

Supplementary Figure S3A

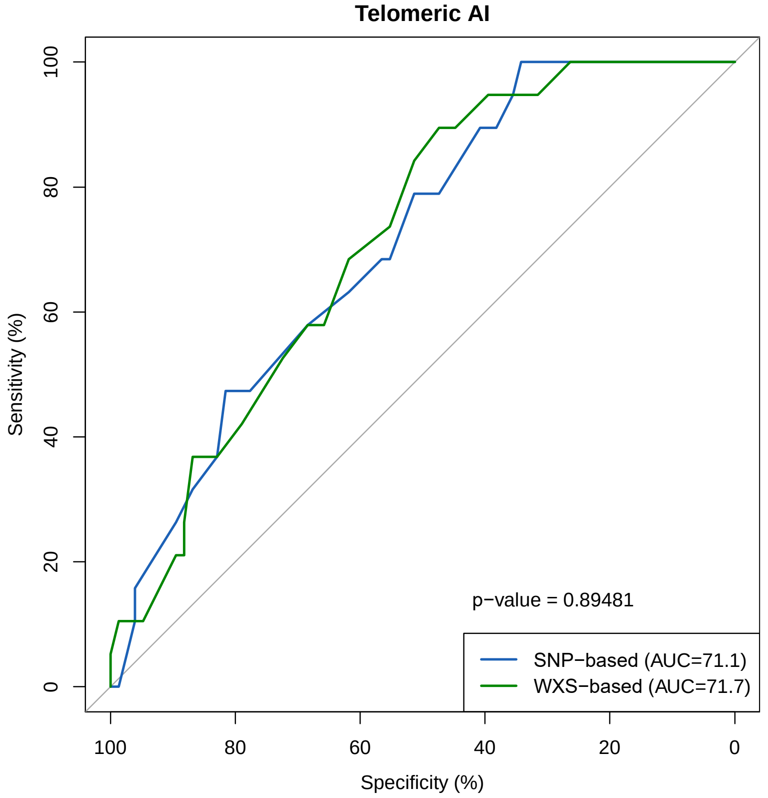

Supplementary Figure S3A: ROC curves showing the ability of the WXS-based and SNP-array based Telomeric Allelic Imbalance score to predict the BRCA1/2 deficiency status in n=95 triple-negative breast cancer patients of the TCGA dataset

Supplementary Figure S3B

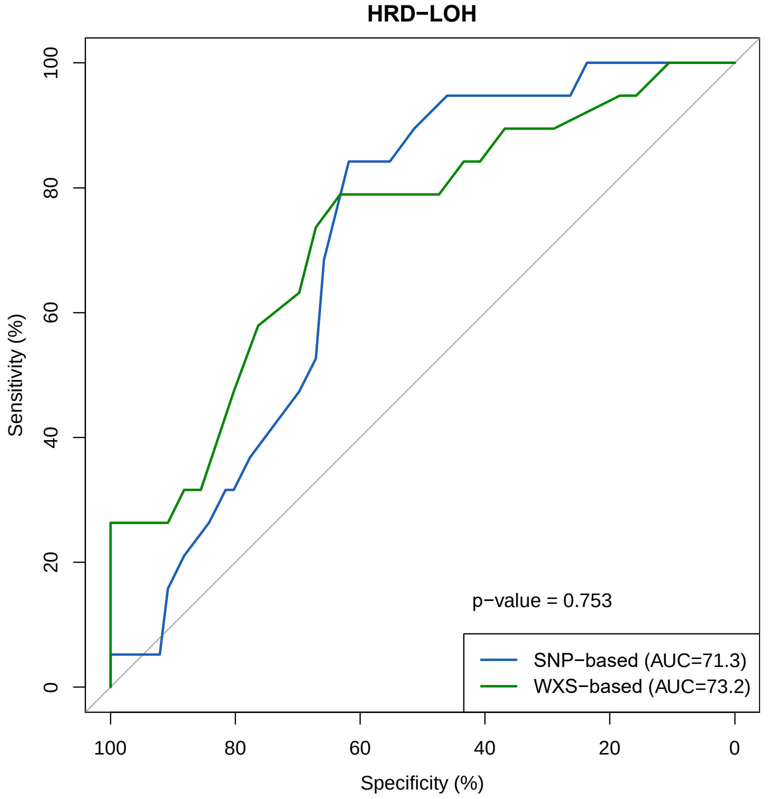

Supplementary Figure 3B: ROC curves showing the ability of the WXS-based and SNP-array based HRD-LOH score to predict the BRCA1/2 deficiency status in n=95 TNBC patients of the TCGA dataset

Supplementary Figure S3C

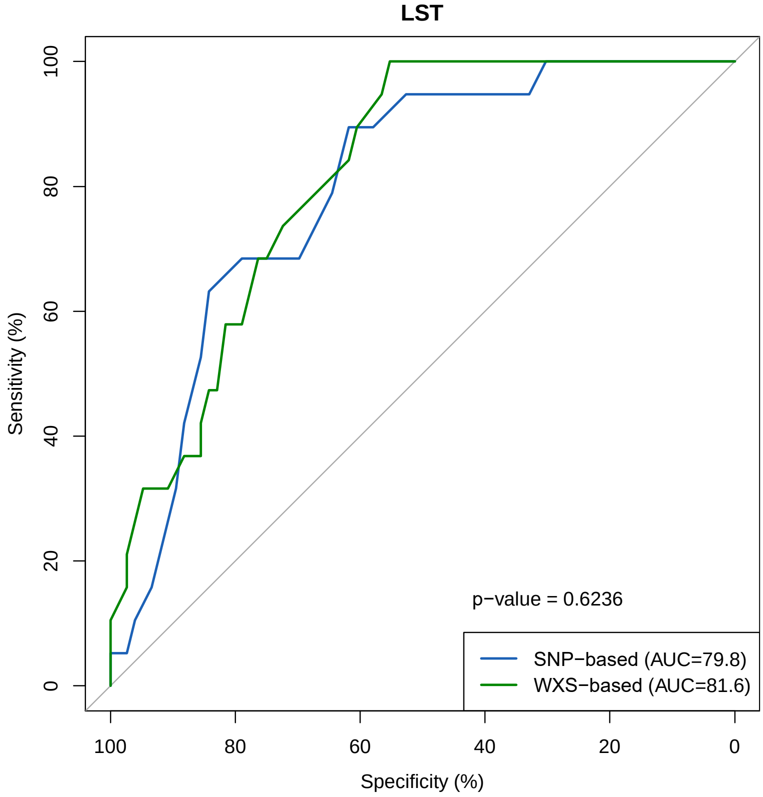

Supplementary Figure 3C: ROC curves showing the ability of the WXS-based and SNP-array based LST score to predict the BRCA1/2 deficiency status in n=95 TNBC patients of the TCGA dataset

Supplementary Figure S3D

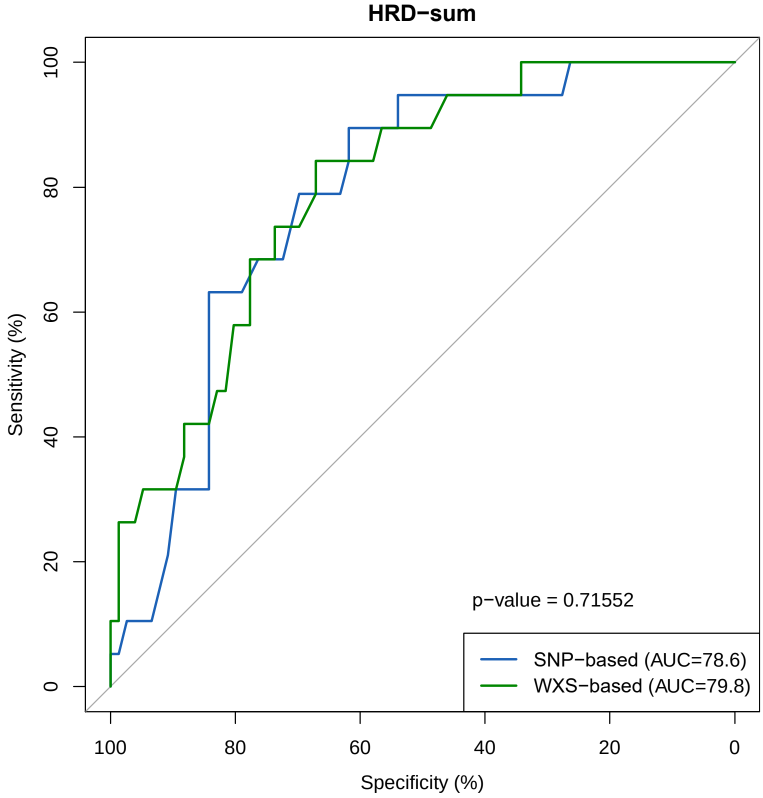

Supplementary Figure 3D: ROC curves showing the ability of the WXS-based and SNP-array based HRD-sum score to predict the BRCA1/2 deficiency status in n=95 TNBC patients of the TCGA dataset

Supplementary Figure S4A:

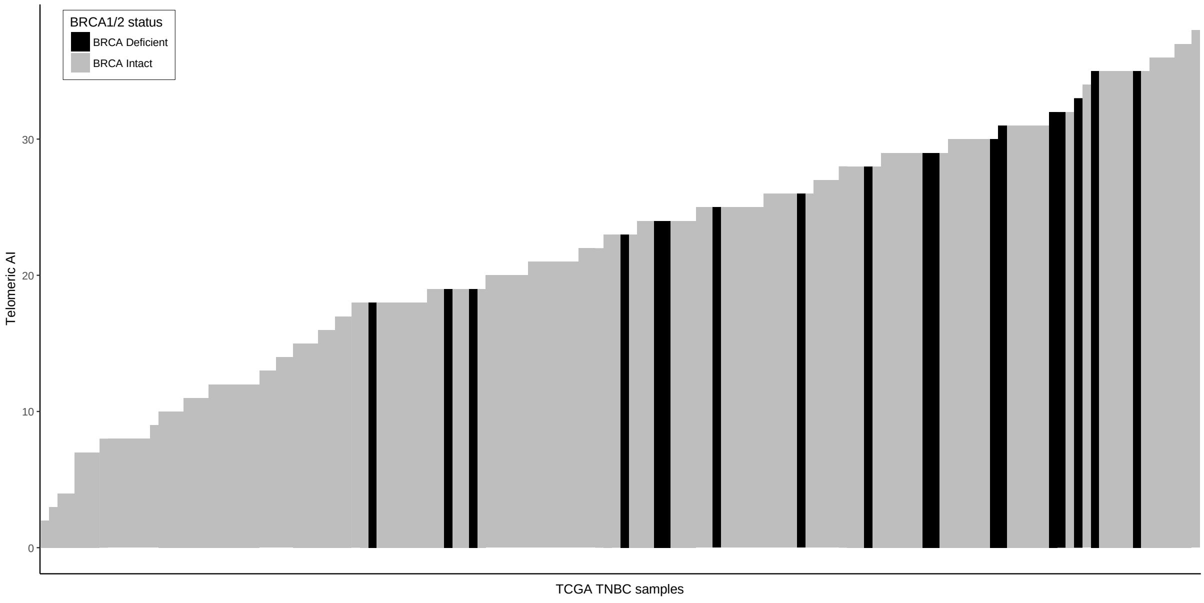

Supplementary Figure S4A: Distribution of the number of Telomeric allelic imbalances in TNBC patients (n=139), the black lines represent the BRCA1/2 deficient samples.

Supplementary Figure S4B:

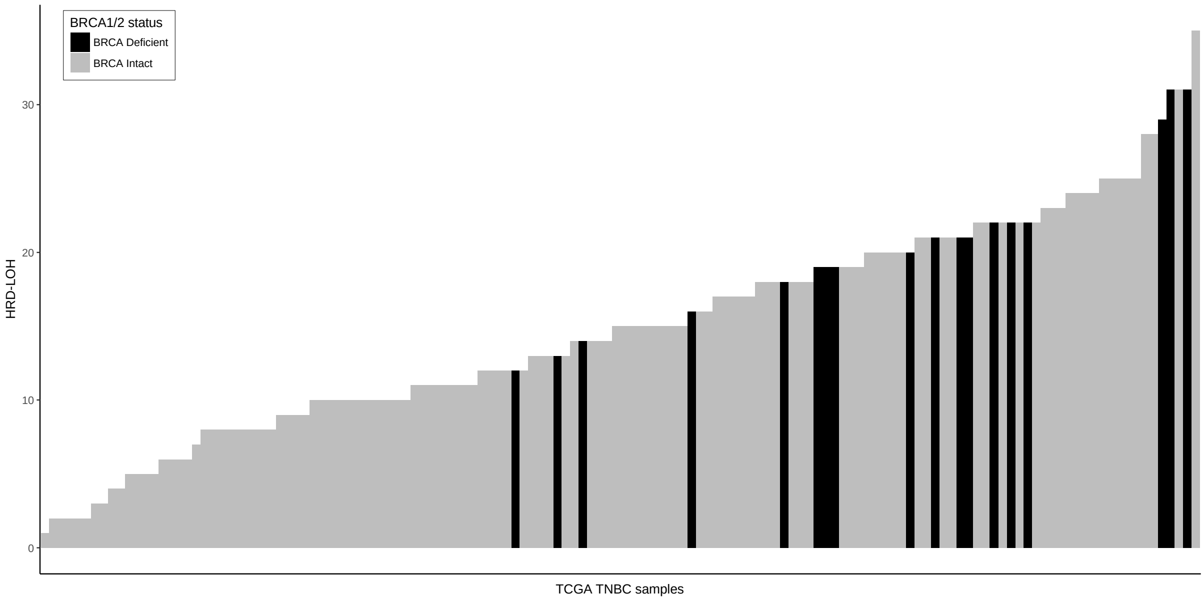

Supplementary Figure S4B: Distribution of the HRD-LOH scores in TNBC patients (n=139), the black lines represent the BRCA1/2 deficient samples.

Supplementary Figure S4C:

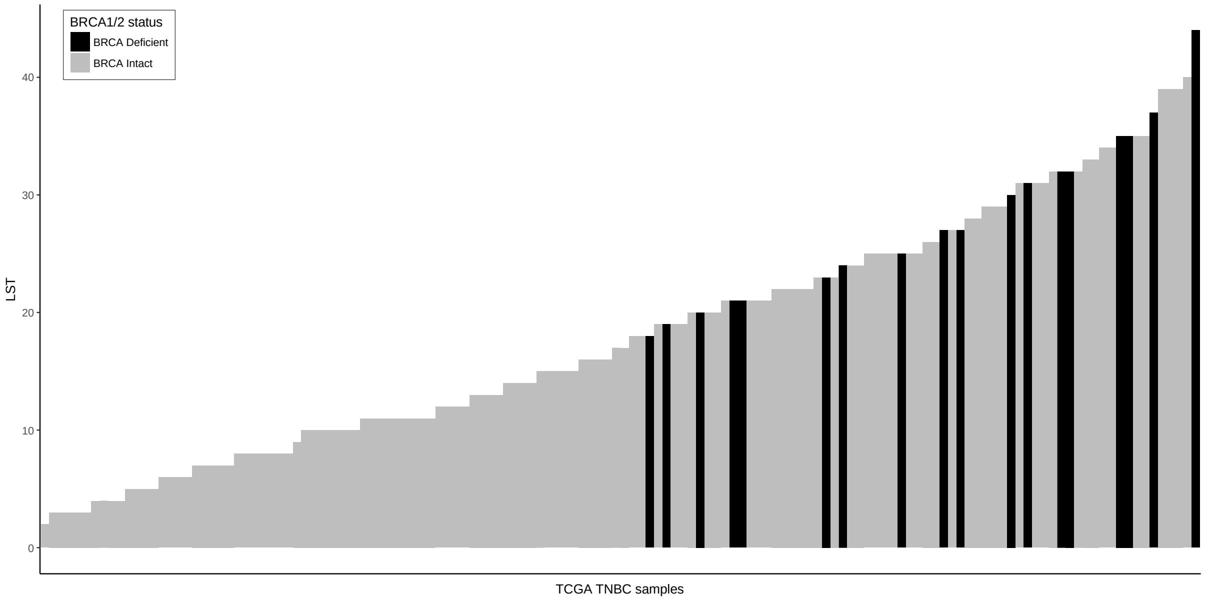

Supplementary Figure S4C: Distribution of the number of Large-Scale Transitions (LSTs) in TNBC patients (n=139), the black lines represent the BRCA1/2 deficient samples.

Supplementary Figure S4D:

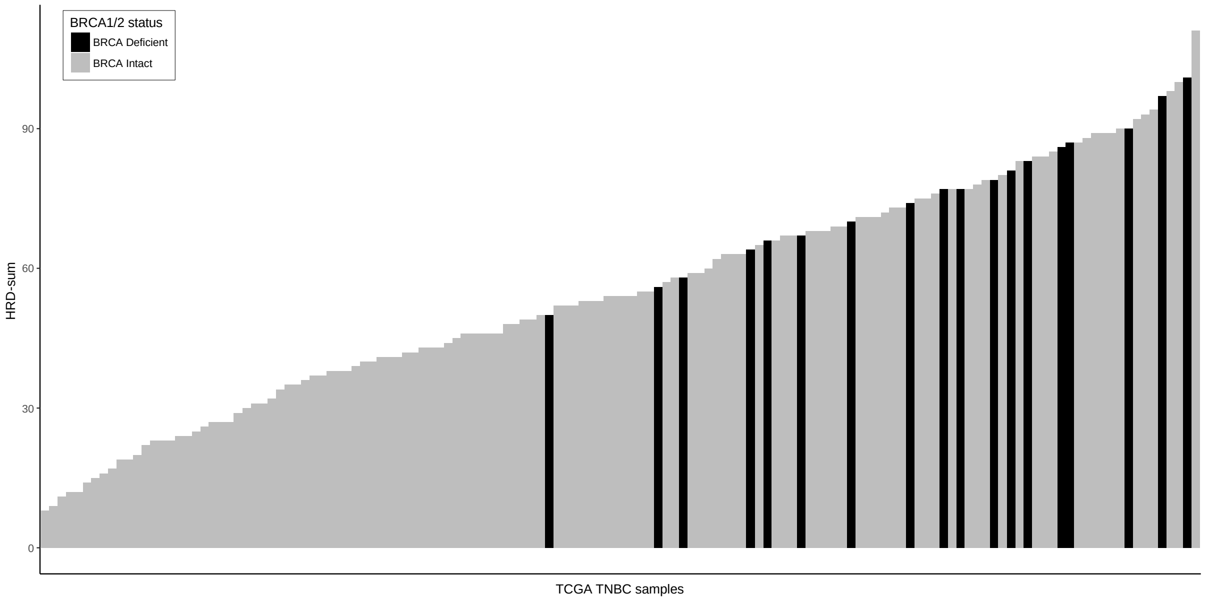

Supplementary Figure S4D: Distribution of HRD-sum values in TNBC patients (n=139), the black lines represent the BRCA1/2 deficient samples.

Supplementary Figure S5

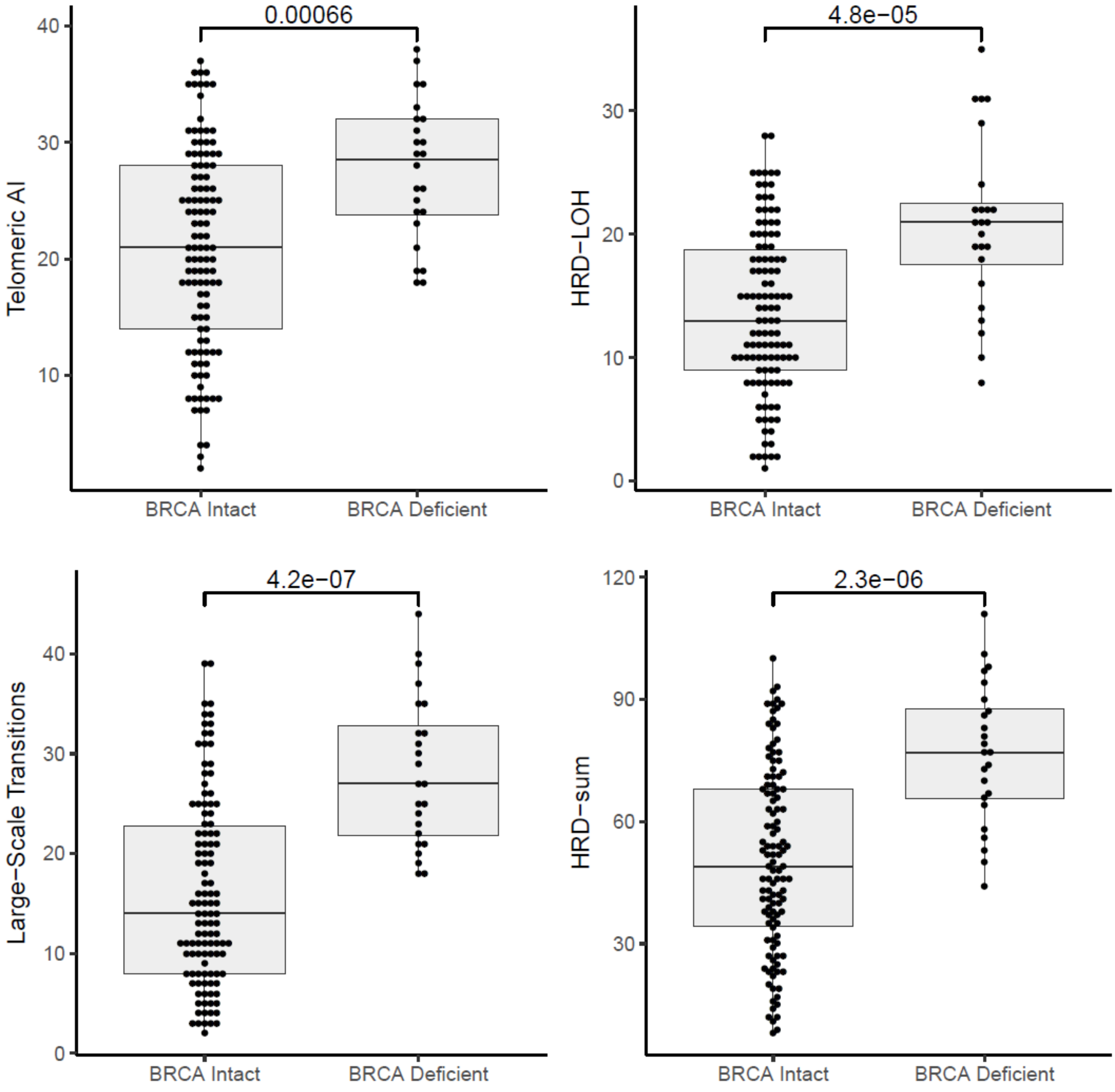

Supplementary Figure S5: Differences between BRCA-deficient and BRCA-intact samples for the WXS-derived genomic scar scores (n=139.)
A sample was classified as BRCA-deficient if (1) there was a deep deletion of BRCA1/2, (2) a germline and a somatic mutation in BRCA1/2 with LOH, or (3) if LOH had co-occurred with promoter methylation in one of the BRCA1/2 genes.

Supplementary Figure S6

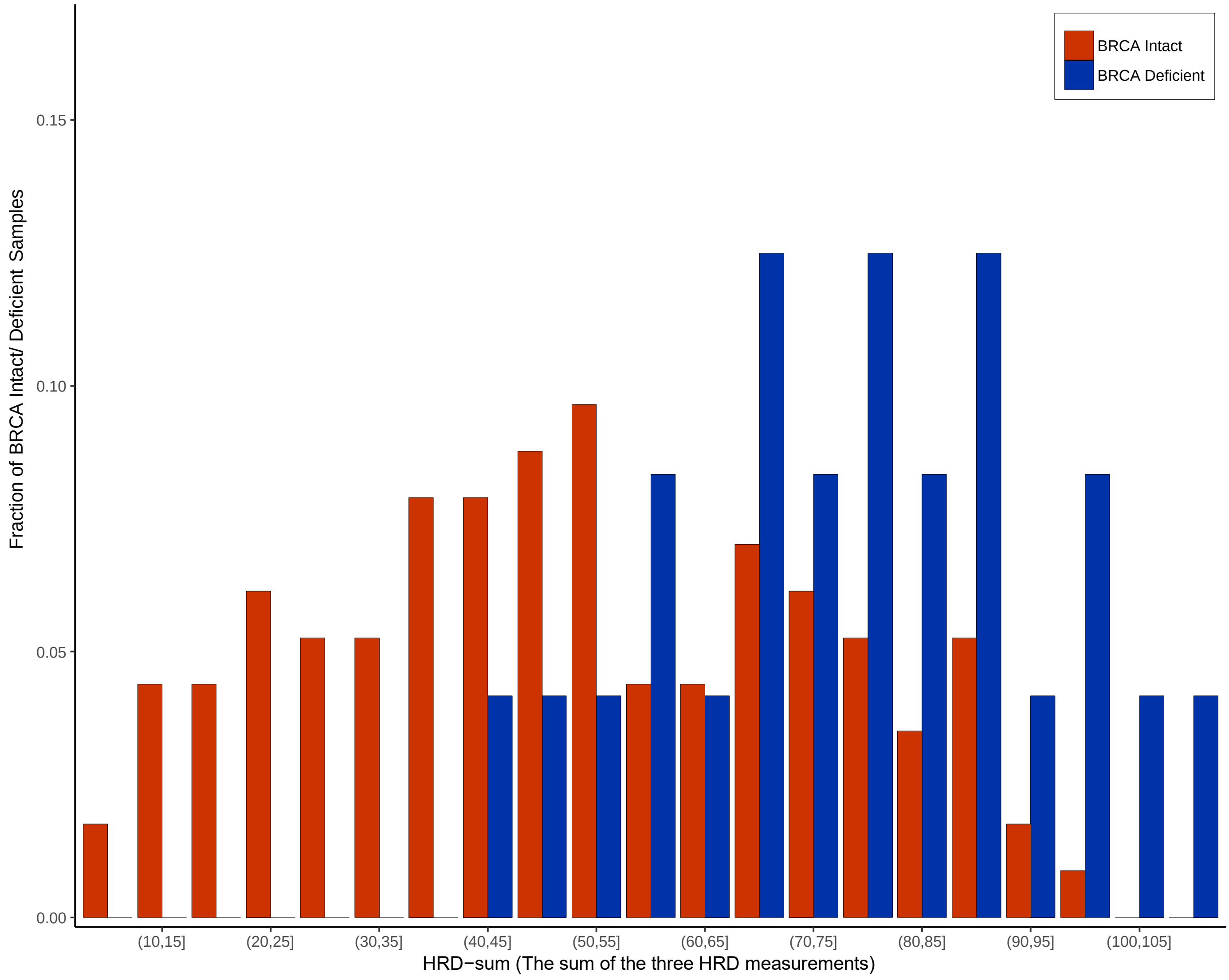

Supplementary Figure S6: The difference of HRD-sum distribution between BRCA Intact and Deficient samples represented in a modified version of Figure 2, where the y-axis represents the fraction of the samples from the BRCA Intact or Deficient group instead of the number of patients

Supplementary Figure S7

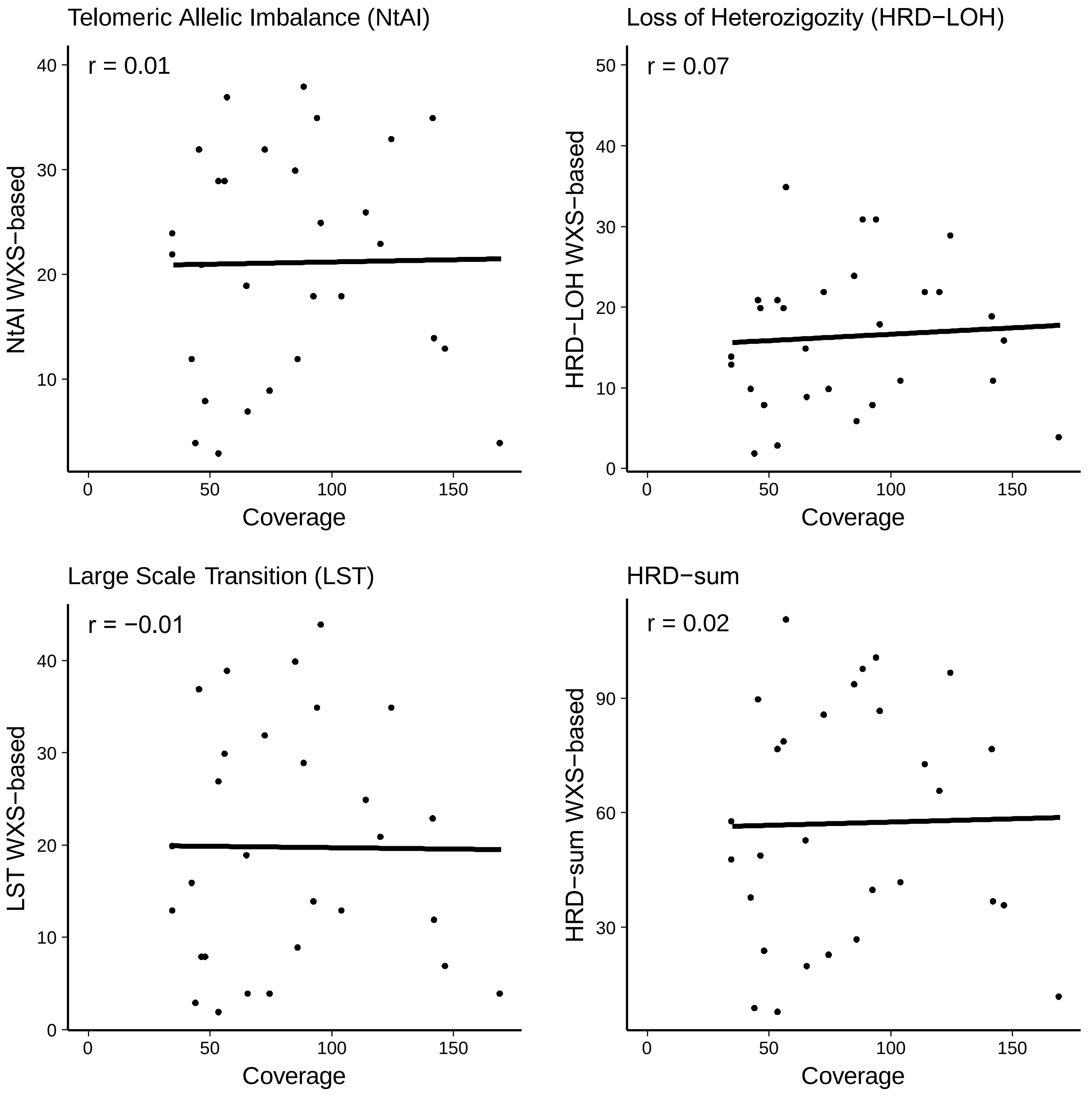

Supplementary Figure S7: The effect of coverage on the individual genomic scar scores, in 15-15 randomly selected BRCA-deficient and BRCA-intact samples. The Pearson correlation between the coverage and the genomic scars were low in each of the cases

Supplementary Figure S8

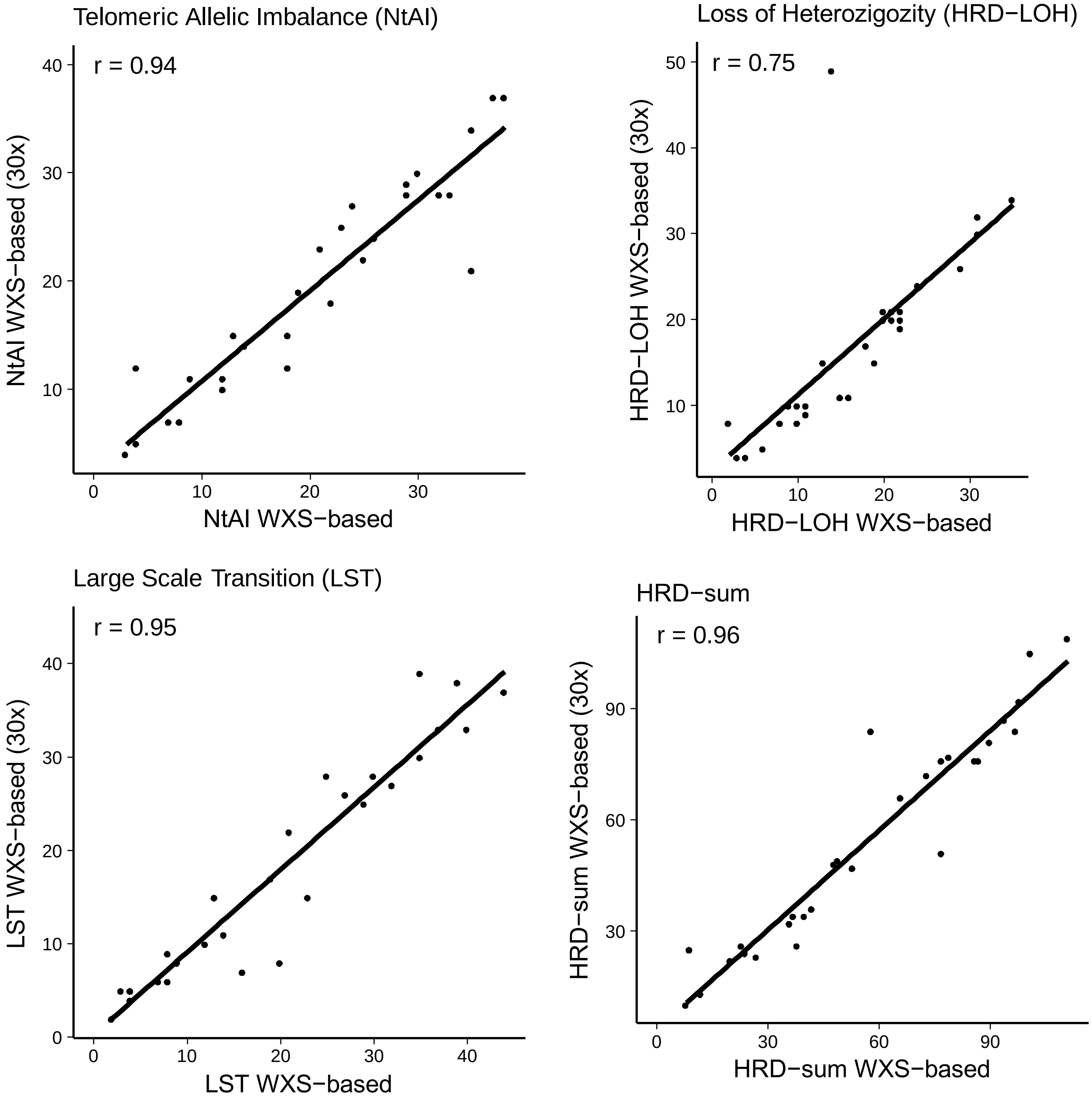

Supplementary Figure S8: The effect of lower coverage (30x) on the genomic scar scores in 15-15 randomly selected BRCA-deficient and BRCA-intact samples. Each of the genomic scar score showed high Pearson-correlation between the 30x (subsampled) and the original samples.

References

1 Abkevich, V. *et al.* Patterns of genomic loss of heterozygosity predict homologous recombination repair defects in epithelial ovarian cancer. *Br. J. Cancer* **107**, 1776-1782, doi:10.1038/bjc.2012.451 (2012).

2 Robin, X. *et al.* pROC: an open-source package for R and S+ to analyze and compare ROC curves. *BMC Bioinformatics* **12**, 77, doi:10.1186/1471-2105-12-77 (2011).

3 Pedersen, B. S. & Quinlan, A. R. Mosdepth: quick coverage calculation for genomes and exomes. *Bioinformatics* **34**, 867-868, doi:10.1093/bioinformatics/btx699 (2018).

4 Li, H. *et al.* The Sequence Alignment/Map format and SAMtools. *Bioinformatics* **25**, 2078-2079, doi:10.1093/bioinformatics/btp352 (2009).

Supplementary Table S1

PatientID	HRD.LOH_WXS	Telomeric.AI_WXS	LST_WXS	HRDsum_WXS	HRD.LOH_SNP	Telomeric.AI_SNP	LST_SNP	HRDsum_SNP	BRCA.status	BRCA1germline	BRCA2germline	BRCA1somatic	BRCA2somatic	BRCA1_LOH	BRCA2_LOH	BRCA1methylation (beta-value)	BRCA2methylation (beta-value)
TCGA-A1-A0SK	8	18	14	40	10	17	18	45	BRCA Intact	0	0	0	0	1	1	0.03	0.01
TCGA-A1-A0SO	31	35	35	101	23	33	29	85	BRCA1 Deficient (germline mutation and LOH)	1	0	1	0	1	0	0.03	0.01
TCGA-A1-A0SP	10	11	10	31	11	9	3	23	BRCA Intact	0	0	0	0	1	1	0.03	0.01
TCGA-A2-A04P	22	36	31	89	21	34	28	83	BRCA Intact	0	0	0	1	1	0	NA	NA
TCGA-A2-A04Q	9	17	16	42					BRCA Intact	0	0	0	0	1	0	NA	NA
TCGA-A2-A04T	29	33	35	97	29	32	28	89	BRCA1 Deficient (germline mutation and LOH)	1	0	0	0	1	0	NA	NA
TCGA-A2-A04U	17	30	16	63	12	28	19	59	BRCA Intact	0	0	0	0	1	1	NA	NA
TCGA-A2-A0CM	9	20	17	46					BRCA Intact	0	0	0	0	1	1	NA	NA
TCGA-A2-A0D0	14	29	28	71	25	34	28	87	BRCA Intact	0	0	0	0	1	0	NA	NA
TCGA-A2-A0D2	19	31	31	81					BRCA1 Deficient (germline mutation and LOH)	1	0	0	0	1	1	NA	NA
TCGA-A2-A0ST	1	8	7	16					BRCA Intact	0	0	0	0	0	0	0.02	0.01
TCGA-A2-A0SX	12	17	10	39	31	14	14	59	BRCA Intact	0	0	0	0	1	1	0.02	0.01
TCGA-A2-A0T0	18	27	34	79	21	22	36	79	BRCA Intact	0	1	0	1	0	0	0.02	0.01
TCGA-A2-A0T2	15	23	11	49	10	13	8	31	BRCA Intact	0	0	0	0	1	1	0.03	0.01
TCGA-A2-A0YE	17	19	10	46	30	23	11	64	BRCA Intact	0	0	0	0	1	1	0.04	0.01
TCGA-A2-A1G6	2	7	3	12	0	0	0	0	BRCA Intact	0	0	0	0	0	0	0.02	0.01
TCGA-A2-A3XS	15	18	13	46	19	22	15	56	BRCA Intact	0	0	0	0	1	1	0.02	0.01
TCGA-A2-A3XT	10	28	21	59	18	24	33	75	BRCA Intact	0	0	0	0	1	1	0.03	0.01
TCGA-A2-A3XU	9	7	4	20	7	7	5	19	BRCA Intact	0	0	0	0	0	0	0.02	0.01
TCGA-A2-A3XX	8	8	3	19	4	18	25	47	BRCA Intact	0	0	0	0	1	0	0.02	0.01
TCGA-A7-A0DA	15	35	39	89	25	38	32	95	BRCA Intact	0	0	0	0	1	0	NA	NA
TCGA-A7-A26F	11	23	18	52	13	13	13	39	BRCA Intact	0	0	0	0	1	0	0.03	0.01
TCGA-A7-A26G	11	12	15	38					BRCA Intact	0	0	0	0	1	1	0.02	0.01
TCGA-A7-A26I	22	21	7	50	24	29	8	61	BRCA Intact	0	0	0	0	1	1	0.02	0.01
TCGA-A7-A4SD	15	19	19	53	17	23	13	53	BRCA Intact	0	0	0	0	1	1	0.02	0.01
TCGA-A7-A4SE	24	27	16	67	36	28	17	81	BRCA Intact	0	0	0	0	1	1	0.04	0.01
TCGA-A7-A6VV	18	26	10	54					BRCA Intact	0	0	0	0	1	1	0.02	0.01

PatientID	HRD.LOH_WXS	Telomeric.AI_WXS	LST_WXS	HRDsum_WXS	HRD.LOH_SNP	Telomeric.AI_SNP	LST_SNP	HRDsum_SNP	BRCA.status	BRCA1germline	BRCA2germline	BRCA1somatic	BRCA2somatic	BRCA1_LOH	BRCA2_LOH	BRCA1methylation (beta-value)	BRCA2methylation (beta-value)
TCGA-A7-A6VW	16	19	21	56					BRCA1 Deficient (somatic mutation and LOH)	0	0	1	0	1	1	0.03	0.01
TCGA-A7-A6VY	22	18	24	64					BRCA1 Deficient (LOH and promoter hypermethhylation)	0	0	0	0	1	1	0.46	0.01
TCGA-A8-A07O	6	12	9	27	7	10	6	23	BRCA Intact	0	0	0	0	0	1	NA	NA
TCGA-A8-A08R	23	35	35	93	29	36	27	92	BRCA Intact	0	0	0	0	1	1	NA	NA
TCGA-A8-A09X	5	24	14	43					BRCA Intact	0	0	0	0	1	0	NA	NA
TCGA-AC-A2BK	22	22	11	55	28	28	21	77	BRCA Intact	0	0	0	0	0	1	0.03	0.01
TCGA-AC-A2QH	13	22	13	48	15	18	16	49	BRCA Intact	0	0	0	0	1	1	0.03	0.01
TCGA-AC-A2QJ	10	10	3	23	2	7	5	14	BRCA Intact	0	0	0	0	0	1	0.02	0.01
TCGA-AC-A6IW	11	24	10	45					BRCA Intact	0	0	0	0	1	1	0.03	0.01
TCGA-AC-A7VC	7	10	5	22					BRCA Intact	0	0	0	0	1	1	0.02	0.01
TCGA-AN-A04D	11	21	26	58	13	21	27	61	BRCA Intact	0	0	0	0	0	1	NA	NA
TCGA-AN-A0AL	22	23	21	66	23	25	30	78	BRCA1 Deficient (germline mutation and LOH)	1	0	0	1	1	0	NA	NA
TCGA-AN-A0AR	25	28	32	85	32	29	30	91	BRCA Intact	0	0	0	0	1	0	NA	NA
TCGA-AN-A0AT	25	36	39	100	25	27	23	75	BRCA Intact	0	0	0	1	1	0	NA	NA
TCGA-AN-A0G0	11	21	11	43	10	40	19	69	BRCA Intact	0	0	0	0	0	1	NA	NA
TCGA-AN-A0XU	24	30	40	94	22	31	31	84	BRCA Intact	0	0	1	0	1	0	0.75	0.01
TCGA-AO-A0J2	24	30	35	89	26	33	33	92	BRCA Intact	0	0	0	0	1	0	NA	NA
TCGA-AO-A0J4	10	21	22	53	20	23	20	63	BRCA Intact	0	0	1	0	1	0	NA	NA
TCGA-AO-A0J6	22	26	25	73	22	27	32	81	BRCA Intact	1	0	0	0	1	1	NA	NA
TCGA-AO-A124	31	38	29	98	30	38	23	91	BRCA Intact	1	0	0	1	1	1	0.02	0.01
TCGA-AO-A128	23	35	25	83					BRCA Intact	0	0	0	0	1	0	0.03	0.01
TCGA-AO-A1KR	8	18	18	44	11	21	13	45	BRCA Intact	0	0	1	0	1	1	0.02	0.01
TCGA-AQ-A04J	15	20	24	59					BRCA Intact	0	0	0	0	1	1	NA	NA
TCGA-AQ-A54N	20	21	8	49	19	23	4	46	BRCA Intact	0	0	0	0	1	1	0.03	0.01
TCGA-AR-A0TS	13	30	25	68					BRCA Intact	0	0	0	0	1	1	NA	NA
TCGA-AR-A0TU	35	37	39	111	41	35	38	114	BRCA Intact	0	0	0	0	1	1	0.64	0.01

PatientID	HRD.LOH_WXS	Telomeric.AI_WXS	LST_WXS	HRDsum_WXS	HRD.LOH_SNP	Telomeric.AI_SNP	LST_SNP	HRDsum_SNP	BRCA.status	BRCA1germline	BRCA2germline	BRCA1somatic	BRCA2somatic	BRCA1_LOH	BRCA2_LOH	BRCA1methylation (beta-value)	BRCA2methylation (beta-value)
TCGA-AR-A0U1	25	34	31	90	36	29	32	97	BRCA Intact	0	0	0	0	0	1	NA	NA
TCGA-AR-A1AI	18	26	21	65	28	32	28	88	BRCA Intact	0	0	0	1	1	0	0.02	0.01
TCGA-AR-A1AQ	10	20	8	38					BRCA Intact	0	0	0	0	1	1	0.02	0.01
TCGA-AR-A1AR	16	13	7	36	11	13	7	31	BRCA Intact	0	0	0	0	1	1	0.03	0.01
TCGA-AR-A1AY	11	14	12	37	21	12	10	43	BRCA Intact	0	0	0	0	1	1	0.04	0.01
TCGA-AR-A256	20	30	21	71	21	29	17	67	BRCA Intact	0	0	0	0	1	0	0.03	0.01
TCGA-AR-A2LH	6	11	6	23					BRCA Intact	0	0	0	0	1	1	0.02	0.01
TCGA-AR-A2LR	20	26	22	68	19	29	12	60	BRCA Intact	0	0	0	0	1	1	0.02	0.01
TCGA-AR-A5QQ	5	7	5	17					BRCA Intact	0	0	0	0	0	1	0.02	0.01
TCGA-B6-A3ZX	8	18	11	37					BRCA Intact	0	0	0	0	1	1	0.02	0.01
TCGA-B6-A400	25	28	23	76	25	32	24	81	BRCA Intact	0	0	0	0	1	1	0.03	0.01
TCGA-B6-A402	11	25	12	48	8	24	10	42	BRCA Intact	0	0	0	0	1	0	0.02	0.01
TCGA-B6-A409	12	12	10	34					BRCA Intact	0	0	0	0	1	1	0.02	0.01
TCGA-BH-A0AV	21	27	25	73					BRCA Intact	0	0	0	0	1	1	NA	NA
TCGA-BH-A0B3	6	10	16	32					BRCA Intact	0	0	0	0	0	0	0.04	0.01
TCGA-BH-A0B9	11	18	13	42	16	16	12	44	BRCA Intact	0	0	0	0	1	1	0.03	0.01
TCGA-BH-A0BG	20	35	12	67	22	28	20	70	BRCA Intact	0	0	0	0	1	0	NA	NA
TCGA-BH-A0BL	19	35	23	77	25	23	23	71	BRCA1 Deficient (germline mutation and LOH)	1	0	0	0	1	1	NA	NA
TCGA-BH-A0BW	15	25	22	62	24	20	21	65	BRCA Intact	0	0	0	0	1	1	NA	NA
TCGA-BH-A0E0	8	16	8	32	27	19	9	55	BRCA Intact	0	0	0	0	1	1	0.03	0.01
TCGA-BH-A0RX	2	12	11	25					BRCA Intact	0	0	0	0	0	0	0.03	0.01
TCGA-BH-A0WA	19	26	25	70	25	34	30	89	BRCA1 Deficient (somatic mutation and LOH)	0	0	1	0	1	1	0.05	0.01
TCGA-BH-A18G	4	4	4	12	2	4	5	11	BRCA Intact	0	0	0	0	1	0	NA	NA
TCGA-BH-A18V	28	31	33	92	37	34	27	98	BRCA Intact	0	0	0	0	1	0	NA	NA
TCGA-BH-A1F6	12	29	14	55	19	17	20	56	BRCA Intact	0	0	0	0	1	0	0.02	0.01
TCGA-BH-A1FC	10	20	11	41	16	32	30	78	BRCA Intact	0	0	0	0	0	1	0.02	0.01
TCGA-BH-A42U	10	11	6	27	0	0	0	0	BRCA Intact	0	0	0	0	0	1	0.02	0.01

PatientID	HRD.LOH_WXS	Telomeric.AI_WXS	LST_WXS	HRDsum_WXS	HRD.LOH_SNP	Telomeric.AI_SNP	LST_SNP	HRDsum_SNP	BRCA.status	BRCA1germline	BRCA2germline	BRCA1somatic	BRCA2somatic	BRCA1_LOH	BRCA2_LOH	BRCA1methylation (beta-value)	BRCA2methylation (beta-value)
TCGA-C8-A12V	5	15	7	27					BRCA Intact	0	0	0	0	1	1	NA	NA
TCGA-C8-A131	2	8	4	14					BRCA Intact	0	0	0	0	1	0	NA	NA
TCGA-C8-A1HJ	25	37	26	88	22	27	21	70	BRCA Intact	0	0	0	0	1	0	0.02	0.01
TCGA-C8-A26X	10	18	12	40					BRCA Intact	0	0	0	0	1	1	0.02	0.01
TCGA-C8-A27B	21	31	25	77	29	29	19	77	BRCA Intact	0	0	0	0	1	1	0.02	0.01
TCGA-C8-A3M7	2	4	3	9	4	3	4	11	BRCA Intact	0	0	0	0	1	0	0.03	0.01
TCGA-D8-A13Z	16	31	28	75					BRCA Intact	0	0	0	0	1	1	NA	NA
TCGA-D8-A142	12	31	20	63	36	26	17	79	BRCA Intact	0	0	0	0	0	1	NA	NA
TCGA-D8-A143	15	16	10	41	30	25	24	79	BRCA Intact	0	0	0	0	1	1	NA	NA
TCGA-D8-A147	18	25	44	87	23	28	35	86	BRCA1 Deficient (germline mutation and LOH)	1	0	0	0	1	1	NA	NA
TCGA-D8-A1JF	6	15	8	29	16	19	18	53	BRCA Intact	0	0	0	0	1	0	0.02	0.01
TCGA-D8-A1JG	17	18	11	46					BRCA Intact	0	0	0	0	1	1	0.03	0.01
TCGA-D8-A1JL	12	28	27	67	31	34	32	97	BRCA1 Deficient (somatic mutation and LOH)	0	0	0	0	1	1	0.3	0.01
TCGA-D8-A1XK	18	29	22	69	26	30	26	82	BRCA Intact	0	0	0	0	1	0	0.02	0.01
TCGA-D8-A1XQ	22	32	32	86	19	36	29	84	BRCA1 Deficient (germline mutation and LOH)	1	0	0	0	1	0	0.02	0.01
TCGA-D8-A27F	31	24	19	74	31	29	24	84	BRCA1 Deficient (somatic mutation and LOH)	0	0	0	0	1	1	0.55	0.01
TCGA-D8-A27M	14	24	20	58	28	20	24	72	BRCA1 Deficient (somatic mutation and LOH)	0	0	1	0	1	1	0.03	0.01
TCGA-E2-A14N	21	30	32	83					BRCA1 Deficient (germline mutation and LOH)	1	0	0	0	1	0	0.03	0.01
TCGA-E2-A14R	21	36	27	84	22	34	32	88	BRCA Intact	0	0	0	0	1	1	NA	NA
TCGA-E2-A14X	6	18	6	30	6	25	29	60	BRCA Intact	0	0	0	0	1	0	NA	NA
TCGA-E2-A150	15	20	19	54	20	23	16	59	BRCA Intact	0	0	0	0	1	1	NA	NA
TCGA-E2-A158	17	29	29	75	21	27	35	83	BRCA Intact	0	0	0	0	1	1	NA	NA
TCGA-E2-A159	10	10	6	26					BRCA Intact	0	0	0	0	1	1	NA	NA
TCGA-E2-A1AZ	14	24	14	52	16	31	25	72	BRCA Intact	0	0	0	0	1	1	0.02	0.01

PatientID	HRD.LOH_WXS	Telomeric.AI_WXS	LST_WXS	HRDsum_WXS	HRD.LOH_SNP	Telomeric.AI_SNP	LST_SNP	HRDsum_SNP	BRCA.status	BRCA1germline	BRCA2germline	BRCA1somatic	BRCA2somatic	BRCA1_LOH	BRCA2_LOH	BRCA1methylation (beta-value)	BRCA2methylation (beta-value)
TCGA-E2-A1B6	8	19	8	35					BRCA Intact	0	0	0	0	1	0	0.03	0.01
TCGA-E2-A1L7	21	25	32	78	15	29	31	75	BRCA Intact	0	0	0	0	1	1	0.06	0.01
TCGA-E2-A1LG	10	9	4	23	8	9	2	19	BRCA Intact	0	0	0	0	0	0	0.02	0.01
TCGA-E2-A1LH	18	31	31	80					BRCA Intact	0	0	0	0	1	0	0.02	0.01
TCGA-E2-A1LI	14	24	15	53	32	30	27	89	BRCA Intact	0	0	0	0	1	1	0.02	0.01
TCGA-E2-A1LL	13	19	22	54	12	23	23	58	BRCA Intact	0	0	0	0	1	1	0.02	0.01
TCGA-E2-A574	19	23	15	57	18	24	17	59	BRCA Intact	0	0	0	0	0	0	0.02	0.01
TCGA-E9-A5FL	22	26	23	71	23	27	28	78	BRCA Intact	0	0	0	0	1	0	0.02	0.01
TCGA-EW-A1OV	15	15	11	41					BRCA Intact	0	0	0	0	1	0	0.01	0.01
TCGA-EW-A1P1	2	8	5	15	0	0	0	0	BRCA Intact	0	0	0	0	0	0	0.03	0.01
TCGA-EW-A1P4	20	24	25	69	25	28	37	90	BRCA Intact	0	0	0	0	1	1	0.02	0.01
TCGA-EW-A1P7	5	12	7	24					BRCA Intact	0	0	0	0	0	1	0.01	0.01
TCGA-EW-A1P8	23	29	8	60	20	30	9	59	BRCA Intact	0	0	0	0	1	1	0.02	0.01
TCGA-EW-A1PH	10	25	19	54	12	17	15	44	BRCA Intact	0	0	0	0	0	1	0.02	0.01
TCGA-EW-A3U0	19	35	33	87	29	34	24	87	BRCA Intact	0	0	0	0	1	1	0.04	0.01
TCGA-GI-A2C9	21	32	37	90	26	31	33	90	BRCA1 Deficient (somatic mutation and LOH)	0	0	0	0	1	1	0.68	0.01
TCGA-GM-A2DB	8	14	13	35					BRCA Intact	0	0	0	0	1	0	0.02	0.01
TCGA-GM-A2DD	3	13	15	31					BRCA Intact	0	0	0	0	1	1	0.02	0.01
TCGA-GM-A2DF	13	19	18	50					BRCA1 Deficient (somatic mutation and LOH)	0	0	0	0	1	1	0.19	0.01
TCGA-GM-A2DH	12	25	29	66					BRCA Intact	0	0	0	0	0	0	0.01	0.01
TCGA-GM-A2DI	8	8	3	19					BRCA Intact	0	0	0	0	0	0	0.02	0.01
TCGA-GM-A3XL	20	29	30	79	24	29	32	85	BRCA1 Deficient (somatic mutation and LOH)	0	0	0	0	1	0	0.73	0.01
TCGA-HN-A2NL	28	32	24	84	25	33	24	82	BRCA Intact	0	0	0	0	1	1	0.02	0.01
TCGA-LL-A441	13	22	17	52					BRCA Intact	0	0	0	0	1	1	0.02	0.01
TCGA-LL-A5YO	14	21	11	46					BRCA Intact	0	0	0	0	1	0	0.09	0.01
TCGA-LL-A73Y	24	28	20	72					BRCA Intact	0	0	0	1	1	0	0.03	0.01

PatientID	HRD.LOH_WXS	Telomeric.AI_WXS	LST_WXS	HRDsum_WXS	HRD.LOH_SNP	Telomeric.AI_SNP	LST_SNP	HRDsum_SNP	BRCA.status	BRCA1germline	BRCA2germline	BRCA1somatic	BRCA2somatic	BRCA1_LOH	BRCA2_LOH	BRCA1methylation (beta-value)	BRCA2methylation (beta-value)
TCGA-LL-A740	3	3	2	8	3	3	3	9	BRCA Intact	0	0	0	0	0	0	0.02	0.01
TCGA-OL-A5D6	8	8	8	24	7	6	7	20	BRCA Intact	0	0	0	0	1	0	0.02	0.01
TCGA-OL-A5D7	17	25	21	63	20	25	14	59	BRCA Intact	0	0	0	0	1	1	0.02	0.01
TCGA-OL-A5RW	21	29	27	77	22	25	31	78	BRCA1 Deficient (somatic mutation and LOH)	0	0	0	0	1	0	0.85	0.01
TCGA-OL-A66I	9	19	15	43	16	21	20	57	BRCA Intact	0	0	0	0	1	1	0.02	0.01
TCGA-OL-A66P	10	12	16	38	6	8	20	34	BRCA Intact	0	0	0	0	1	1	0.02	0.01
TCGA-OL-A6VO	19	29	20	68					BRCA Intact	0	0	0	0	1	1	0.02	0.01
TCGA-OL-A97C	4	2	5	11					BRCA Intact	0	0	0	0	0	0	0.03	0.01
TCGA-S3-AA10	18	25	34	77					BRCA Intact	0	0	0	0	1	1	0.03	0.03

Supplementary Table S1: Detailed table of the BRCA1/2 mutation status of the TCGA TNBC samples (germline, somatic mutations, loss-of-heterozigosity state and methylation), the SNP-array and the WXS-based genomic scar scores.
